# The successful use of autologous skin in management of guidewire‐induced distal coronary perforation

**DOI:** 10.1002/ccr3.966

**Published:** 2017-05-12

**Authors:** Ruiwei Guo, Lixia Yang

**Affiliations:** ^1^ Department of CardiologyKunming General Hospital of Chengdu Military CommandKunmingYunnanChina

**Keywords:** Angiography, carotid artery disease, complications, intervention, percutaneous coronary intervention

## Abstract

The successful use of autologous skin to management may provide a useful and widely applicable method for dealing with the troublesome complication of guidewire‐induced coronary perforation.

## Introduction

Percutaneous coronary intervention (PCI) is the primary way to treat coronary heart disease and is a procedure that is always at risk for serious complications 1. Among them, coronary artery perforation is rare and sometimes lethal. Some reports have shown that the incidence of coronary perforations ranges from 0.1% to 4.0%. In most cases, the perforation involves the pericardium, which may lead to cardiac tamponade. The mortality rate of coronary perforations has been reported to be about 7–40% 2. Two‐year registry included 12,900 cases of PCI showed that the coronary artery perforation was more common in female patients, older age patients, patients with diabetes, and patients with lesion complexity.

The severity of perforation is classified into three angiographic types: type I, the development of an extraluminal crater without extravasation, and the incidence of pericardial tamponade was 8%; type II, a pericardial or myocardial blush without contrast jet extravasation; and type III, extravasation through a perforation or cavity spilling into an anatomic cavity, and 63% of patients developed pericardial tamponade and the mortality rate was about 19% 3. The type of distal coronary artery perforation with a coronary guidewire depends on its anatomic position. Guidewire‐induced distal coronary artery perforation is a rare but potentially fatal complication, due to the fact that it is not usually discovered during PCI and causes acute pericardial tamponade after the procedure. There are currently several methods of managing coronary artery perforation, but they always cause a conflict with antithrombotic therapy after PCI 4. Herein, we introduce an expeditious way to deal with guidewire‐induced distal coronary artery perforation using autologous skin.

## Methods

This operation is composed of four key processes. First, a guidewire must be used to access and traverse the perforations of the coronary artery. Second, a small amount of skin is excised from the puncture point. The size of skin should be determined according to the size of the target vessel and the area of perforation, and the layer of the skin deployed mainly is epidermal layer. Next, the skin is pierced using the end of the guidewire and fixed onto the guidewire. The skin is then pushed along the guidewire by a balloon to the narrowed area of the coronary artery containing the perforation. Finally, the guidewire is withdrawn into the balloon and then contrast‐injected to determine whether the coronary artery is blocked. Antithrombotic drugs do not need to be discontinued after the operation. We have successfully managed six such cases between 2011 and 2015; 2‐year follow‐up data showed no complications or sequelae after this management approach. The study protocol conforms to the ethical guidelines of the 1975 Declaration of Helsinki as reflected in a priori approval by our institutional human research committee.

## Results

**Figure 1:**
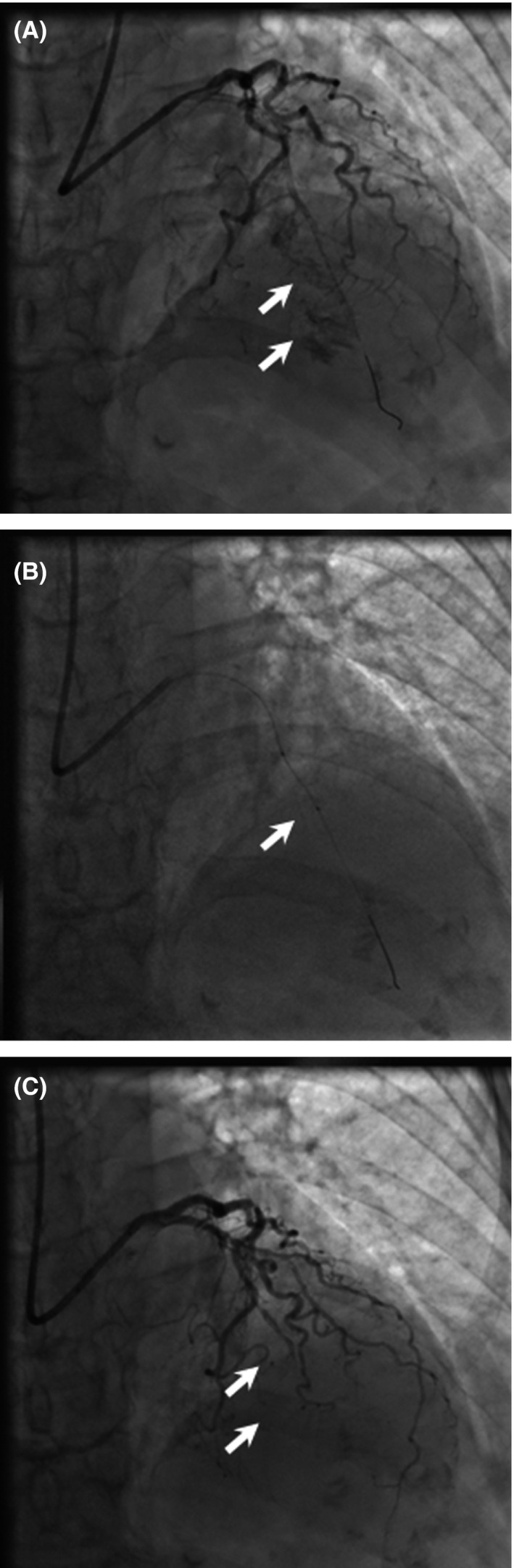
Angiography indicates successful treatment of distal LAD perforation. (A) White arrows highlight the area of contrast extravasation at perforation site. (B) White arrows indicate the balloon used to push the skin. (C) White arrows show that there is no further communication of contrast into distal perforation site.

One of our patients was a 67‐year‐old female who presented with clinical symptoms suggestive of unstable angina. She had a history of hypertension and hyperlipidemia. Routine blood, urine and stool tests, liver and kidney function, and chest radiography were normal. The resting electrocardiogram (ECG) demonstrated minor T‐wave flattening in the precordial leads without other specific abnormalities. Coronary angiography and possible coronary revascularization were considered. Coronary angiography showed total occlusion in the middle segment of the left anterior descending (LAD) coronary artery. After discussing treatment with the patient, it was decided to perform PCI of the LAD artery. The POLIT 50 guidewire (hydrophilic‐coated guidewire) reached the distal vessel after several attempts. After expansion with a 2.75‐mm‐diameter balloon at 8 atm, contrast agent overflow was seen at the distal left LAD artery (Fig. 1A). Fortunately, the patient did not experience discomfort and had normal blood pressure with a steady heart rate. Although balloon occlusion was used within an hour to apply pressure, rapid outward bleeding continued for more than two hours. Because there was narrowing in the middle of the LAD artery, we planned to block this kind of distal coronary perforation by autologous skin according to this above process. Autologous skin was introduced by balloon along the guidewire to the narrowed place of the coronary artery with perforation (Fig. 1B). Then, the guidewire was withdrawn into the balloon, and serial angiography over the next 10 min demonstrated complete and persistent occlusion of the distal branch (Fig. 1C).

**Figure 2:**
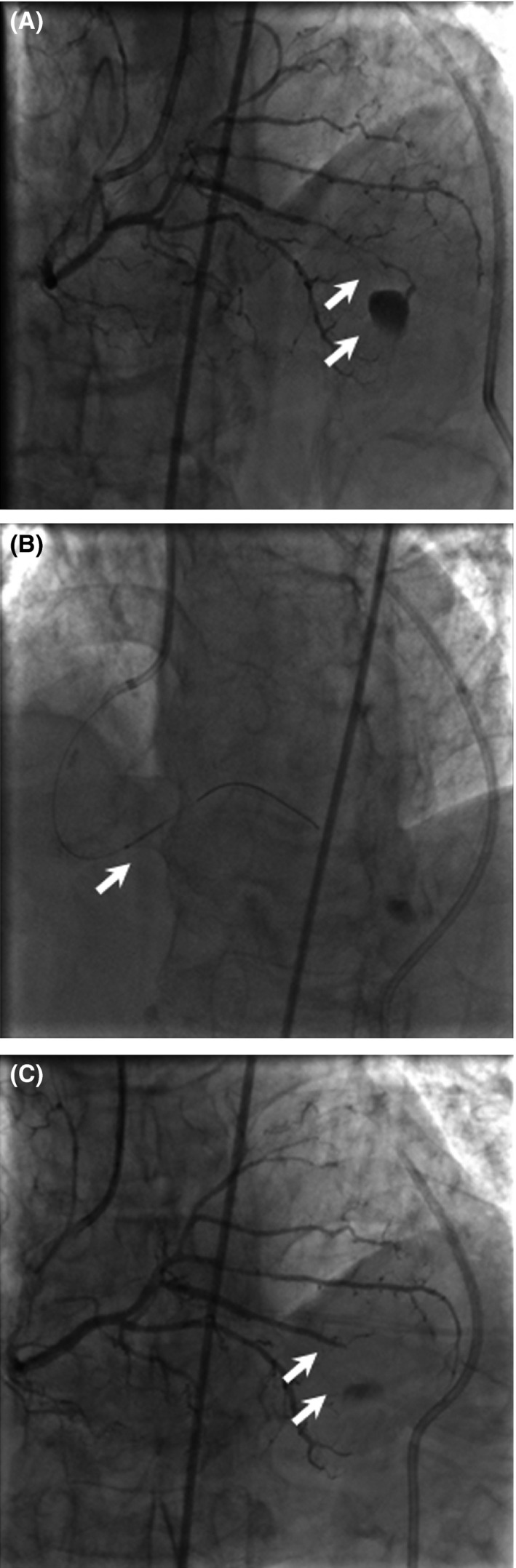
Angiography indicates successful management of distal PDA perforation. (A) White arrows highlight the area of contrast extravasation at perforation site. (B) White arrows indicate the balloon used to push the skin. (C) There is no further communication of contrast into distal perforation site.

Another patient was a 73‐year‐old male who was referred to our center following a non‐Q‐wave myocardial infarction. His comorbidities included hypertension, hyperlipidemia, and diabetes. After angiography, a stent was implanted successfully in the posterior descending artery (PDA). The patient complained of chest discomfort shortly after returning to the ward, associated with tachycardia and hypotension. Echocardiography indicated an acute cardiac tamponade. After pericardiocentesis, the patient was immediately returned to the catheterization laboratory. Angiography showed that contrast agent overflow was seen at the distal PDA (Fig. 2A). The guidewire reached the coronary perforation, and the autologous skin was introduced by balloon to the narrow portion before the PDA perforation. Then, the guidewire was withdrawn into the balloon (Fig. 2B), and serial angiography demonstrated complete and persistent occlusion of the distal branch (Fig. 2C).

## Discussion

The incidence of coronary perforation during PCI is low, but there are some risk factors that increase coronary perforation rate, such as female gender, older age, diabetes, and lesion complexity 5. In addition, use of a cutting balloon or rotational atherectomy device is also associated with an increased risk of coronary perforation 6. Complications of coronary perforation vary greatly, from clinical insignificance to hemodynamic collapse and death, depending on the management approach. The management of coronary perforation often includes heparin reversal, discontinuation of glycoprotein IIb/IIIa inhibitors, platelet transfusion, pericardiocentesis, and emergency cardiac surgery. Additional treatment strategies include prolonged balloon inflation, covered stents, intracoronary administration of thrombin, and surgical evacuation 7, 8, 9. But patients that received intracoronary administration of thrombin often do not undergo standard antithrombotic treatment, which would pose a risk to patients undergoing PCI, particularly those with implanted stents 10. Unwise use of gelfoam, gelatin microspheres, or polyvinyl‐alcohol increases the risk of peripheral artery occlusion. The availability for injection of these do not always exist in laboratories performing PCI. We must spend some time to get these materials, which will increase mortality of patients. However, after the use of autologous skin to block the distal coronary perforation, we did not need to cease antithrombotic treatment, as there was no foreign matter in the coronary artery. Meanwhile, reversal of heparin is not necessary. Therefore, the question of coronary thrombosis did not arise. And it is very ease to get the autologous skin. Six cases have been managed, and successful rate is 100%. Follow‐up data showed that the long‐ and short‐term safety and effectiveness of autologous skin are excellent, and thus, this approach may be useful for all cases. However, there are limitations to this method. This approach can only be applied to the distal coronary perforation and requires that the guidewire must quickly reach the target vessel. This approach to management may be simpler and more effective than some of the other techniques described and may provide a useful and widely applicable method for dealing with the troublesome complication of guidewire‐induced coronary perforation.

## Authorship

RG: involved in the implementation of the operation and the summary of the data. LY: involved in the suggestion and future advices for this operation.

## Conflicts of Interest

None declared.
